# Computer-Aided Diagnostics of Heart Disease Risk Prediction Using Boosting Support Vector Machine

**DOI:** 10.1155/2021/3152618

**Published:** 2021-12-23

**Authors:** Ebenezer Owusu, Prince Boakye-Sekyerehene, Justice Kwame Appati, Julius Yaw Ludu

**Affiliations:** Department of Computer Science, University of Ghana, Legon, Accra, Ghana

## Abstract

Heart diseases are a leading cause of death worldwide, and they have sparked a lot of interest in the scientific community. Because of the high number of impulsive deaths associated with it, early detection is critical. This study proposes a boosting Support Vector Machine (SVM) technique as the backbone of computer-aided diagnostic tools for more accurately forecasting heart disease risk levels. The datasets which contain 13 attributes such as gender, age, blood pressure, and chest pain are taken from the Cleveland clinic. In total, there were 303 records with 6 tuples having missing values. To clean the data, we deleted the 6 missing records through the listwise technique. The size of data, and the fact that it is a purely random subset, made this approach have no significant effect for the experiment because there were no biases. Salient features are selected using the boosting technique to speed up and improve accuracies. Using the train/test split approach, the data is then partitioned into training and testing. SVM is then used to train and test the data. The C parameter is set at 0.05 and the linear kernel function is used. Logistic regression, Nave Bayes, decision trees, Multilayer Perceptron, and random forest were used to compare the results. The proposed boosting SVM performed exceptionally well, making it a better tool than the existing techniques.

## 1. Introduction

Heart disease refers to a variety of conditions that affect the heart from contamination to genetic deficiencies and blood-vessel diseases. These defects are among the topmost causes of deaths globally for all races. In 2016, about 28.2 million adults in the United State were diagnosed with this condition [1] and in 2015 nearly 634000 people died [2] making it the foremost cause of deaths. According to the American Heart Association, a nonprofit organization that funds cardiovascular medical research, one American has a heart attack every 40 seconds [3]. Per the data, there are 720,000 new cases of heart attacks and 335,000 chronic attacks in the United States each year. The form of heart or cardiovascular disease- (CVD-) related morbidity and mortality has been rather fascinating in Sub-Saharan Africa, an area thought to have the world's youngest population. Sub-Saharan Africa remained the only region in the globe where heart disease-related fatalities increased between 1990 and 2013 [4]. The World Health Organization (WHO), for example, has listed heart disease as one of the top two causes of death in Ghana, after diarrheal infections [5]. In 2008, heart disease was the leading cause of death in Ghana among all noncommunicable diseases (NCDs) and the major cause of institutional deaths, accounting for 14.5 percent of all deaths reported [6].

Traditionally, a patient's need to know the status of his heart condition was based on the doctor's view. Before doing any test, the doctor will likely perform a few physical checks and interrogate the patient to examine his medical history, regardless of the severity of the cardiac problem. With the exception of blood tests and chest X-rays, any heart disease diagnosis may include the involvement of an electrocardiogram (ECG), which records electrical signals that aid in the discovery of anomalies in the heart's rhythm and structure. Holter monitoring echocardiogram, stress test, Cardiac Catheterizations, Cardiac Computerized Tomography (CT) Scan, and Cardiac Magnetic Resonance Imaging (MRI) are some of the other therapies. A Holter monitor is a small, wearable device that captures an ECG during a 24- to 72-hour period. Holter monitoring detects heart rhythm abnormalities that are not at all noticeable on a standard ECG. The echocardiogram consists of an ultrasound image of the chest and detailed images of the heart's construction and function. A stress test, often known as a treadmill test or an exercise test, is used by doctors to determine how well the patient's heart can endure workload. The patient will engage in some physical activity or take drugs to raise their heart rate for this test. After that, the actual examination and various photographs of the heart are taken to analyze the underlying reality. In case you ask your doctor if you have heart disease, the standard procedure is for him to assess the likelihood based on risk factors. Age, diabetes, smoking, high blood pressure, being male, and cholesterol are all significant risk factors. According to previous studies, nearly half of those who had coronary attacks had two risk factors: being male and being over 60[7]. As a result, it is incredibly exciting that technology has enabled early diagnosis and risk assessment straightforward before people develop the disease.

**Figure 1:**
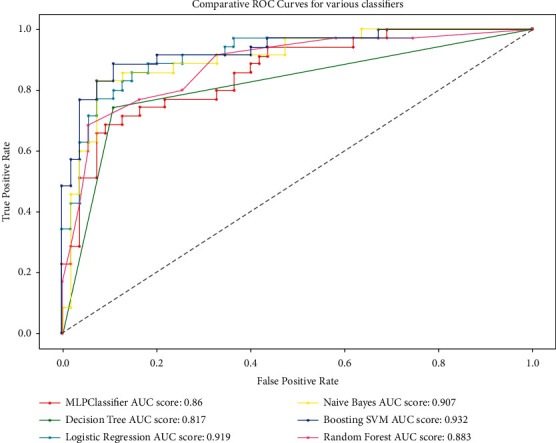
Comparative ROC of various classifiers.

Owing to the increased risk of heart disease and the fact that current research forecasts computer-assisted treatments, this study aims to suggest two novel approaches to the problem. To begin, we offer a better algorithm that enhances diagnosis, and then we explain how the proposed method is unquestionably superior to earlier proposed techniques by demonstrating the technique's real implementation. Tables 1, 2, 3, and 4 and Figure 1 demonstrate unequivocally that the suggested method is superior to earlier proposed methods. The remaining part of the study is structured as follows: previous related studies and their challenges are presented in Section 2. The proposed technique and how data is preprocessed as well as previous algorithms employed to solve the problem are discussed in Section 3. The result of the study is then discussed in Section 4. The conclusions are finally drawn in Section 5.

## 2. Related Studies

Several methods have been used to predict the risks of getting heart disease. Genetic algorithms, for example, have been used in a variety of applications. According to [8], the neurofuzzy system combines the capabilities of neuroadaptive capability and fuzzy logic reasoning for the prediction of the heart disease risk level. The algorithms are generally used for weight optimization when training the model, but there is a serious drawback. Genetic algorithms do not guarantee an optimal solution; hence, the weight optimization may not be completely accurate. In comparison to SVM, Naive Bayes, decision tree, and random forest and genetic algorithms are more complicated to implement and require a large number of parameters to be set in order to achieve a result that is close to optimal. As a result, for small datasets like the Cleveland utilized in this investigation, the genetic algorithm is not appropriate.

The Iterative Dichotomiser 3 (ID3) algorithm, a type of decision tree building algorithm [9], is a relatively simple algorithm that has proven to be effective in other areas but has the drawback of only handling categorical data, so it cannot be used in Cleveland, which is plagued by missing values. If the sample data tested is tiny, this approach is prone to overfitting. As a result, it cannot be used for this research.

Deep neural networks [10], which have shown greater performance in prediction, were also excluded from this study because what is learned with deep neural nets is difficult to comprehend. Furthermore, because learning is progressive, deep neural nets require a large amount of data to train the learning algorithms [11]. When compared to random forest, logistic regression, Nave Bayes, neural networks, and decision trees, the proposed boosting SVM algorithm utilized in this study performed well. On small datasets, these solution approaches are among the best-performing algorithms, and they are also a lot easier to grasp.

Miranda et al. [12] used the Naive Bayes algorithm to forecast this health concern and looked at the related risk levels for adults in their study. In this study, blood and urine test results from the clinical laboratory were used as training datasets. The difficulty with this study is that the authors failed to explore ECG and echocardiography analysis, both of which are crucial in detecting cardiovascular diseases, and the accuracy of 80% obtained is comparably poor. Again, since all the properties in Naive Bayes are expected to be mutually independent, using this predictor to predict heart disease is challenging because finding a collection of predictors that are totally independent of one another is extremely difficult in real life.

In addition, neural networks are widely employed [13, 16]. To predict cardiovascular heart disease, Nandy et al. [14] employed a swarm-artificial neural network. The goal of the research was to increase accuracy. While the study's findings were promising, the accuracy of 95.78% needed to be improved, especially when compared to the study we recommended. Sayad and Halkarnikar [17] proposed a data mining and artificial neural network-based detection approach for cardiac disease. A multilayer perceptron neural network (MLPNN) and a backpropagation algorithm were used in this investigation. The residual dataset was separated into two parts after preprocessing. The MLPNN with backpropagation approach had a 92% accuracy, which is below average. Kim and Kang [18] developed a neural network-based technique for predicting the risk of heart disease using the Korea National Health and Nutritional Examination Survey (KNHANES-VI) dataset [19]. This method consists of two steps. A feature sensitivity-based feature selection is the first phase, followed by a neural network-based prediction model. 3031 people were judged to be at low risk out of 4146, whereas 1115 were found to be at high risk. Dutta et al. [20] suggested a convolutional neural network for predicting heart disease by classifying clinical data that was highly class-imbalanced. The study's findings, on the other hand, were not encouraging.

While neural networks are gaining popularity and appear to be realistic, they suffer from data overfitting and temporal complexity. When dimensionality is low, neural networks also fail to converge.

For the same reason, the random forest has been employed in various investigations [21]. Javeed et al. [22] used the Cleveland datasets to construct a random search algorithm (RSA) for feature selection and a random forest model for heart failure prediction. To improve the suggested diagnostic system, the grid search method was applied. Two types of testing were conducted to determine the accuracy of the proposed approach. The first trial only builds a random forest model, whereas the second trial builds the specified RSA-based random forest model. The proposed method has a classification accuracy of 93.33%, and that is not really impressive. Jabbar et al. [23] also proposed a random forest-based classification and feature selection by chi-square and genetic algorithm to predict the risk of heart disease on the Cleveland dataset. The proposed technique outperformed other methods such as Naïve Bayes, decision tree, and neural networks. However, the study's accuracy was only 84%, making it worthless for actual deployment. Decision tree prediction for heart disease has also been proposed [24, 25]. Decision trees, on the other hand, do not work well with missing attributes in the Cleveland datasets if they are not treated with considerable attention, making the outcome inaccurate. The use of logistic regression techniques in the prediction of cardiac disorders is very common. For example, Soleimani and Neshati [26] utilized three logistic regression models with 28 features to predict heart disease risk using 711 data from patients with factors such as severe chest pain, back pain, cold chills, shortness of breath, nausea, and vomiting. However, the study's accuracy of 94.9% was not particularly noteworthy.

A Support Vector Machine (SVM) has also become highly popular. The SVM with sequential minimal optimization strategies was investigated in 2015, with prediction accuracies ranging from 82% to 90%, which was not promising. However, new research into SVM algorithms is yielding better results. Harimoorthy and Thangavelu [27], for example, recently used R studio's SVM-radial bias kernel approach to predict heart disease with 98.7% accuracy.

Based on the favorable results with SVM, we were encouraged to do further examination to improve the technique in the proposed study.

## 3. Materials and Methods

### 3.1. Datasets Description

The Cleveland dataset was used in this study. It is a Cleveland Clinic Foundation dataset containing 14 variables related to patients' vital signs in relation to heart disease. The remaining property is used as the target or projected class, and thirteen of the fourteen qualities are used as predictor variables. Sex, age, type of chest pain, serum cholesterol, resting blood pressure, fasting blood sugar, resting maximum heart rate, electrocardiography, and ST segment elevation are among the study's 13 predictor variables. The expected characteristics include exercise-induced angina, depression, slope, thallium test result, number of vessels damaged by fluoroscopy, and diagnosis. There were 303 data sets in total, with 6 missing values. The 303 records were reduced to 297 by deleting the 6 tuples that have missing records through the listwise method. Looking at the large size of the data, and the fact that it is a purely random subset, this method had no significant effect on the rest of the data used for the experiment because there were no biases.  Table 5 contains descriptions of the datasets.

### 3.2. The Proposed Framework

**Figure 2:**
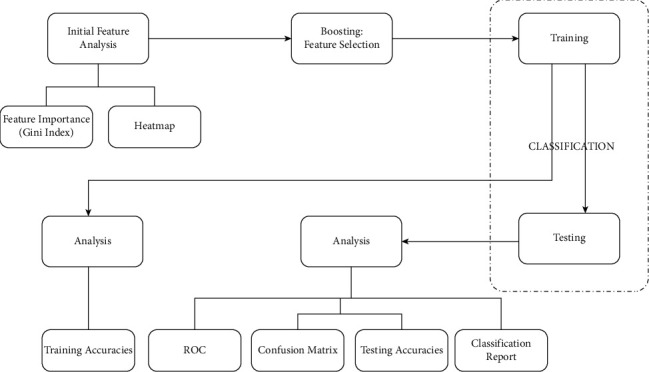
The proposed framework.

The proposed framework for the study is shown in Figure 2.

The framework demonstrates the whole methodology of the proposed technique. The explanations are as follows.

### 3.3. Feature Importance Estimation

**Figure 3:**
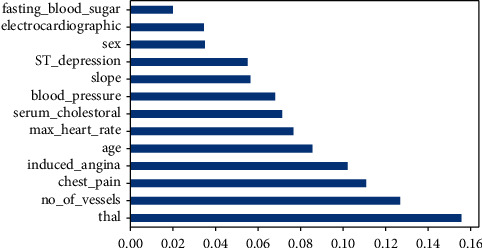
Importance of each feature.

The feature importance score assigns a numerical value to each data feature; the higher the score, the more significant the feature to the output variable. We extracted the top features for the dataset using the Extra Tree Classifier. The amount that each attribute split point improves the performance measure, weighted by the number of observations the node is responsible for, is used to evaluate the relevance of a single decision tree. The purity (Gini index) was used to choose the separation points. The relevance of each attribute is then summed across all decision trees in the model. The Gini index in Algorithm 1 is presented as follows:

The entire method is developed with the goal of maximizing purity in each split. Purity is defined in (1) as the degree to which the groupings are homogeneous:

(1) $$\begin{array}{c} \text{Gini}=1-\underset{j}{\sum }p_{j}^{2}, \end{array}$$

where *p*_*j*_ is the probability of an object being classified to a particular class with label *j* number of times.  Figure 3 shows the degree of importance of each feature.

### 3.4. Feature Correlation Matrix

**Figure 4:**
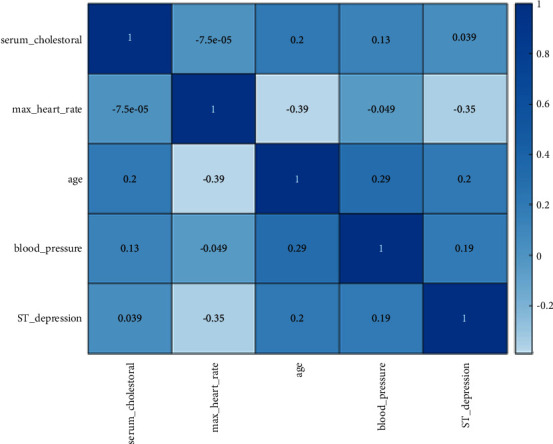
A correlation matrix with heatmap.

A correlation is a term that describes how features are related to one another. The heatmap makes it simple to see which features are most closely associated with the target variable. Using the seaborn library, we created a heatmap of connected features. Pearson's correlation coefficient was used in this study. This correlation evaluates how closely two numerical sequences are positively connected. We plotted Pearson's heatmap to see the correlation of independent variables. By using AdaBoost as feature selection algorithm, only selected features which have correlation above 0.5, taking into consideration absolute values, were selected. The Seaborn functions automatically perform the statistical estimation required to complete operation. The factors in deep blue in Figure 4 show the highest correlation, namely, max. heart rate and age and ST depression and max. heart rate, indicating that both “age” and “max. heart rate” will play a significant role in predicting heart disease.

### 3.5. Boosting SVM Classification

Boosting is an ensemble meta-algorithm that, in essence, removes dataset biases for machine learning algorithms and upgrades weak learners to strong learners. The goal of the boosting strategy is to enhance prediction accuracy. The following is a description of the adaptive boosting algorithm that was used:

Let *p* be denoted by positive and *g* negative samples and let each sample be (*S*_*i*_, *y*_*i*_) where *y* ∈ {±1} represents the corresponding class label. The feature selection algorithm is formulated as follows:

Step 1: initialize the sample distribution by weighting every training sample equally such that the initial weights become *w*_1,*i*_=1/2*p* and *w*_1,*i*_=1/2*g* for *y* = 1 and -1, respectively. For the iteration *t*=1,2,…, *T*, where *T* is the final iteration, execute the following.
Step 2: normalize *w*_*t*,*i*_ ← *w*_*t*,*i*_/∑_*i*=1_^*N*^*w*_*t*,*i*_, where *w*_*t*_ is a probability distribution and *N* is total number of features.
Step 3: train a weak classifier *h*_*t*_ for feature *j*, which uses a single feature. The training error *ξ*_*t*_ is estimated with respect to *w*_*t*_ as stated in the following equation:
  (2) $$\begin{array}{c} \xi_{t}=\underset{r}{\sum }w_{t,i}{\left|h_{t}\left(x_{i}\right)-y_{i}\right|}^{2}. \end{array}$$
Step 4: select the hypothesis *h*_*t*_^1^ with the most discriminating information, that is to say, the hypothesis with the least classification error *ξ*_*t*_^1^, on the weighted samples.
Step 5: compute the weight *ω*_*t*_ that weights *h*_*t*_^1^ by its classification performance as in the following equation:
  (3) $$\begin{array}{c} \omega_{t}=\frac{1}{2}\ln\left[\frac{1}{\xi_{t}^{1}}-1\right]. \end{array}$$
Step 6: the weight distribution is then updated and normalized with the following equation:
  (4) $$\begin{array}{c} w_{t+1,i}\approx w_{t,i}.e^{-\omega_{t}y_{i}h_{t}^{1}\left(S_{t}^{1}\right)}. \end{array}$$
Step 7: the final feature selection hypothesis *H(S)* which is a function of the selected features is denoted by the following equation:
  (5) $$\begin{array}{c} H\left(S\right)=\mathrm{sgn}\left[\sum_{t=1}^{T}\omega_{t}h_{t}^{1}\left(S_{t}^{1}\right)\right]. \end{array}$$

Input the Cleveland training datasets sets, represented by {(*y*_1_, *x*_1_),…, (*y*_*N*_, *x*_*N*_)}. *N*=*a*+*b*; where *a* datasets have *y*_*i*_=+1 and *b* datasets have *y*_*i*_=−1. The *b* datasets represent the 0 attributes of the datasets. The scale parameters *x* and *y* are the feature vectors selected by the AdaBoost algorithm. The maximal margin separating the hyperplane becomes an optimization problem shown in the following equations:

(6) $$\begin{array}{c} w^{T}x+k=0, \end{array}$$

(7) $$\begin{array}{c} \underset{w}{\min}\;\frac{1}{2}\left(w^{T}w\right), \end{array}$$

subject to the constraints in the following equation:

(8) $$\begin{array}{c} y_{i}\left(\left(w^{T}x_{i}\right)+k\right)\geq 1. \end{array}$$

Since *w*^*T*^*x*+*k*=0 and *c*(*w*^*T*^*x*+*k*)=0 define the same plane, *w*, *c* is the regularization parameter. *w*^*T*^(*x*_+_)+*k*=0 and *w*(*x*_−_)+*k*=0, where (*x*_+_) and (*x*_−_) are the respective positive and negative support vectors. The margin is then denoted by the following equation:

(9) $$\begin{array}{c} \frac{w}{\left\|w\right\|}\left(\left(x_{+}\right)-\left(x_{-}\right)\right)=\frac{w^{T}\left(\left(x_{+}\right)-\left(x_{-}\right)\right)}{\left\|w\right\|}=\frac{2}{\left\|w\right\|}. \end{array}$$

The optimal plane is solved by using the convex quadratic programming problem in the following equation:

(10) $$\begin{array}{c} \left\{\begin{array}{c} \underset{w\in R^{P},\xi \in R^{+}}{\min}\frac{1}{2}\left(w^{T}w\right)+c\overset{N}{\underset{i=1}{\sum }}\xi_{t},\\ \text{s.t. }y_{i}\left(\left(w^{T}x_{i}\right)+k\right)\geq 1-\xi_{i},\\ \xi_{i}\geq 0, \end{array}\right. \end{array}$$

for *i*=1,…, *N*, *c*=0.05. The decision boundary of the classifier is expressed as the sum over the support vectors in the following equation:

(11) $$\begin{array}{c} f\left(x\right)=\mathrm{sgn}\left(\overset{N}{\underset{i=1}{\sum }}y_{i}\alpha_{i}Q\left(x_{i},x\right)+b\right), \end{array}$$

where *x*_*i*_ is the support vector data, *α*_*i*_ is the Lagrange multiplier, and *y*_*i*_ is the label of membership class (+1, −1) with *n*=1,2,3,…, *N*. The product *Q*(*x*_*i*_, *x*) represents a linear kernel function, given by the following equation:

(12) $$\begin{array}{c} Q\left(x_{i},x\right)=\varphi \left(x_{i}\right)\varphi \left(x\right). \end{array}$$

The linear kernel function *Q*(*x*_*i*_, *x*) transforms the original data space into a new space with a higher dimension; this includes the transformation function with dot product, *φ*(*x*). The reason is to make transformed data easily separable.

### 3.6. Model Evaluation Metrics

An important component of the study is to assess the performance of the proposed method. This is accomplished by comparing the performance of the proposed technique to that of some standard techniques using some acceptable measures. The confusion matrix, classification report, Receiver Operating Characteristic (ROC) curve, and Area under the Curve (AUC) data were used to evaluate the model's performance. The model's test and training accuracies must also be assessed.

#### 3.6.1. Receiver Operating Characteristic Curve

A Receiver Operating Characteristic curve is a graph that depicts a classification model's performance over all categorization levels. The curve represents a comparison of the True Positive Rate (TPR) and the False Positive Rate (FPR) in the following equations:

(13) $$\begin{array}{c} \text{TPR}=\frac{\text{TP}}{\text{TP}+\text{FN}}, \end{array}$$

(14) $$\begin{array}{c} \text{FPR}=\frac{\text{FP}}{\text{FP}+\text{TN}}, \end{array}$$

where TP, FP, FN, and TN represent true positives, false positives, false negatives, and true negatives, respectively.

#### 3.6.2. Area under the Curve

The Area under the Curve (AUC) is the most well-known quantitative index to describe accuracy.

The AUC is computed as follows:

(15) $$\begin{array}{c} \text{AUC}=\frac{1+\text{TPR}-\text{FPR}}{2}. \end{array}$$

Generally, an area of 1 means a perfect test and area of 0.5 represents a worthless test. The general acceptable interpretation of AUC values is displayed in Table 6.

### 3.7. Comparative Algorithms

#### 3.7.1. Comparing SVM with Boosted SVM

Preliminary experiment was conducted using Support Vector Machine (SVM) and the boosted SVM with the same linear kernel function to determine whether the proposed boosted SVM has significant advantages over the traditional SVM. The results show that the accuracies for SVM and the boosting SVM in terms of training and testing accuracies are 86.83% and 83.41% against 99.92% and 99.75%, respectively. This result is statistically significant (*p* < 0.5). Thus, we follow up to compare the results of the proposed method against Logistic regression, Naïve Bayes, decision tree, Multilayer Perceptron, and random forest which are extensively used in this domain.

#### 3.7.2. Logistic Regression

Logistic regression is the best regression analysis to use when the dependent variable or response variable is binary [28]. It works by combining the input variable (*X*) in a linear form and using coefficients to predict an output variable (*Y*) which is a binary value of 0 or 1. The logistic regression technique models the chance of an outcome based on the individual characteristics or input variables (*X*). It is represented mathematically as follows:

(16) $$\begin{array}{c} \log_{10}\frac{\pi }{1-\pi }=\beta_{0}+\beta_{1}x_{1}+\cdots +\beta_{n}x_{n}, \end{array}$$

where *π* indicates the probability of an event, *β* represents estimated parameter values or regression coefficients associated with the variables via maximum likelihood estimation, and *x* indicates the parameter variables.

#### 3.7.3. Naïve Bayes

A Naive Bayes classifier is a simple probabilistic classifier modelled on the application of Bayes' theorem, with strong (Naive) independence assumptions [29]. Naïve Bayes classifier can be trained very efficiently in the context of supervised learning. The Bayesian rule is given in the following equation:

(17) $$\begin{array}{c} P\left(H|X\right)=\frac{P\left(X|A\right)P\left(H\right)}{P\left(H\right)}. \end{array}$$

From above, *P*(*H|X*) is a conditional probability, that is, the likelihood of event *H* occurring given *X* is true. *P*(*X*) and *P*(*H*) are the probabilities of observing *X* and *H* independently of each other.

#### 3.7.4. Decision Tree

The Gini index, impurity (information gain) approach, which evaluates the degree or chance of a given variable being incorrectly classified when it is randomly chosen, was utilized to compare with the proposed method. The term “information gain” refers to the process of determining which characteristic or attribute provides the most information about a class. The Gini impurity is calculated by summing the probabilities *p*_*i*_, of a class with label *i*, times the probability ∑_*k*≠*i*_1 − *p*_*i*_ of a mistake in categorizing that item. The computation is given in the following equation:

(18) $$\begin{array}{c} \text{Gini}=1-\overset{c}{\underset{i=1}{\sum }}{\left(p_{i}\right)}^{2}, \end{array}$$

where *p*_*i*_ is the probability of an object being classified to a particular class.

#### 3.7.5. Multilayer Perceptron

The Multilayer Perceptron (MLP) network is trained using the backpropagation [30], which uses data to adjust the network's weights and thresholds to minimize the error in its predictions on the training set. First, it computes the total weighted input *x*_*j*_, using the following equation:

(19) $$\begin{array}{c} X_{j}=\sum y_{i}w_{ij}, \end{array}$$

where *y*_*i*_ is the activity level of the *j*-th unit in the previous layer and *w*_*ij*_ is the weight of the connection between the *i*-th and the *j*-th unit. Next, the unit calculates the activity *y*_*j*_ using the sigmoid function.

#### 3.7.6. Random Forest

The training algorithm used is the bagging or the bootstrapping aggregating trees. This creates an ensemble of trees where multiple training sets are generated with replacement, meaning data instance can be repeated. The algorithm is represented as follows.

Given a training set *X*=*x*_1_,…, *x*_*n*_ with a response, *Y*=*y*_1_,…, *y*_*n*_, bagging repeatedly (B times) selects a random sample of the training set and fits trees to these samples:

For *b*=1,…, *B*

- Sample, with replacement, n training examples from *X*, *Y*; call *X*_*b*,_*Y*_*b*_.
- Train a classification tree *f*_*b*_ on *X*_*b*,_*Y*_*b*_.

When training is done, predictions for unseen samples *x*′ are done by determining the average of the predictions from all the individual regression trees on *x*′ as stated in the following equation:

(20) $$\begin{array}{c} \hat{f}=\frac{1}{B}\overset{B}{\underset{b=1}{\sum }}f_{b}\left(x^{\prime }\right). \end{array}$$

The process above depicts the original tree bagging algorithm. Random forest, on the other hand, differs in only one way: its algorithm chooses a random subset of features at each candidate split in the learning process (ensemble learning method that tries to reduce the correlation between estimators in an ensemble by training them on random samples of features rather than the entire feature set), also known as feature bagging. The Gini impurity was employed as the criterion because the random forest is based on decision tree and the study is based on classification.

## 4. Results and Discussion

The results of the study are presented as follows: Table 1 shows the different models' training and testing accuracies and its processing time when run on 4 CPUs), ∼2.2 GHz processor of 8192 MB RAM.  Table 2 shows the confusion matrices and Table 3 shows the classification report.

For each method, the value at the upper left corner is the true positive and the one at the upper right corner is the false positive. The lower right corner is the true negative and the lower left corner is the false negative.

Precision refers to the accuracy with which a judgment is made. The upper row values represent the likelihood of heart illness, whereas the lower row values indicate the likelihood of a decision. The harmonic mean of precision and recall is represented by the F1 score. This is a performance-based statistical measure. The capacity to determine the number of samples that test positive for a specific attribute is known as recall. Figure 1 compares the performance of all of the solution models and Table 4 shows the performances of different methods on the Cleveland dataset. We conducted a one-way ANOVA for the results to find if there is a statistically significant difference between the outcome of the proposed technique result and the others in terms of boosting SVM versus random forest, boosting SVM versus Multilayer Perceptron, boosting SVM versus decision tree, boosting SVM versus Naïve Bayes, and finally boosting SVM versus logistic regression. The analysis of the variances, followed by Tukey simultaneous plot at 95% CI, shows that the corresponding means are significantly different (*p* < 0.5) which demonstrates that boosting SVM is the best. Also, tests for the training speed were conducted and the results again show that there was statistically significant difference between groups (*p*=0.029). A further Tukey post hoc analysis shows that the processing time for the boosting SVM was significantly smaller than all the other techniques after pairing boosting SVM and random forest (*p*=0.041), boosting SVM and Multilayer Perceptron (*p*=0.027), boosting SVM and decision tree (*p*=0.038), boosting SVM and Naïve Bayes (*p*=0.04), and boosting SVM and logistic regression (*p*=0.035). All comparatives show that the boosting SVM methodology is extremely promising.

**Figure 5:**
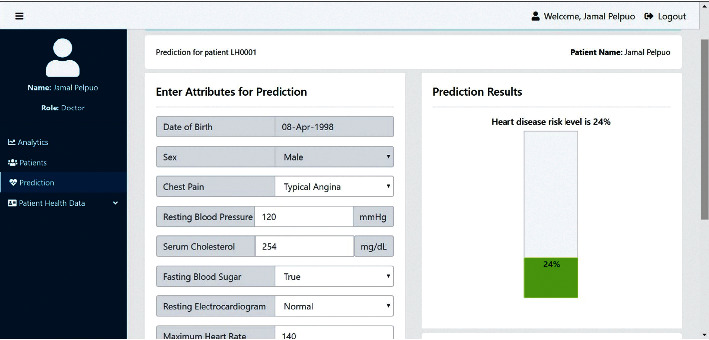
Prediction page showing prediction result for patient with low risk level.

**Figure 6:**
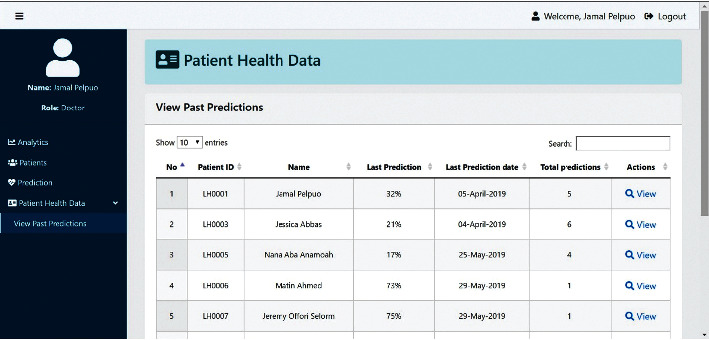
Screen showing prediction history of all patients.

Figures 5 and 6 demonstrate the test application as a proof of concept using the boosting SVM algorithm.

## 5. Conclusion

The study emphasizes the seriousness of cardiac disease and the need of detecting early warning signs. Many machine learning algorithms based on random forest, logistic regression, Multilayer Perceptron, Naive Bayes, and decision trees are being investigated in light of recent studies that call for the automatic detection of dangers. This study proposed a boosting SVM technique to further investigate how to improve prediction accuracy. The technique is based on the Cleveland datasets, which have been utilized successfully and extensively in earlier studies. To reduce misclassification, we preprocessed the data by normalizing it and removing the redundant ones. The feature importance is also computed, which assigns a score to each characteristic in the data; the greater the score, the more relevant the feature to the output variable. Also a heatmap of linked features is produced. The heatmap demonstrates that the most important factors in predicting heart disease are age and maximum heart rates. Finally, classification is performed using the proposed boosting SVM. For the analysis, confusion matrices, classification reports, ROC, and AUC are all used, and the findings reveal that the provided methodologies performed the best. The proposed method has a recognition accuracy of 99.75%, which is much higher than previous studies. The algorithm has now been enacted and has shown to be pretty useful. In the future, we plan to develop a new ensemble model that combines SVM and AdaBoost to improve accuracy and speed, as well as releasing the app on both Android and iOS.

## Figures and Tables

**Algorithm 1:**
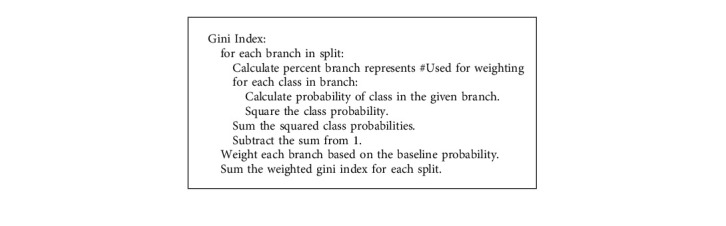
Gini index computation.

**Table 1:** Comparative performance of the training and testing accuracies of methods.

Method	Accuracy		
	Training (%)	Testing (%)	Testing time (s)
Random forest	100	83.33	3.0
Multilayer Perceptron	75.36	80.0	5.8
Decision tree	92.15	83.33	4.0
Naïve Bayes	82.13	85.5	3.2
Logistic regression	84.06	84.44	4.5
Boosting SVM	99.92	99.75	2.1

**Table 2:** Comparative confusion matrices of different methods.

Method	Confusion matrix	
Random forest	47	8
	7	28

Multilayer Perceptron	46	9
	9	26

Decision tree	48	7
	8	27

Naïve Bayes	47	8
	5	30

Logistic regression	45	10
	4	31

Boosting SVM	51	4
	2	33

**Table 3:** Comparing classification report on test data.

Method	Precision	Recall	F1-score	Support
Random forest	0.87	0.85	0.86	55
	0.78	0.80	0.79	35

Multilayer Perceptron	0.84	0.84	0.84	55
	0.74	0.74	0.74	35

Decision tree	0.86	0.87	0.86	55
	0.79	0.77	0.78	35

Naïve Bayes	0.90	0.85	0.88	55
	0.79	0.82	0.82	35

Logistic regression	0.92	0.82	0.87	55
	0.76	0.89	0.82	35

Boosting SVM	0.94	0.87	0.90	55
	0.82	0.89	0.85	35

**Table 4:** Performances of different methods on Cleveland datasets.

Author	Method	Accuracy (%)
Mirza et al. [31]	RBFSVM	87.114
Amen et al. [32]	Logistics regression	82
Sajja et al. [33]	SVM	92–94
Waris & Koteeswaran [34]	Novel KNN	93
Gupta et al. [35]	Naive Bayes	88.16
Saini et al. [36]	Hybrid classifier with weighted voting (HCWV)	82.54
Abdeldjouad et al. [37]	GFS-logicboost-C	94.17
Motarwar et al. [38]	AdaBoost	80.32
Alotaibi [39]	Decision tree	93.19
Gupta et al. [40]	Ensemble of Naïve Bayes, AdaBoost, and boosted tree	87.97
Proposed method	Boosting SVM	99.92

**Table 5:** Description of the attributes.

No	Attribute	Description	Ranges
1	Age	Ages of patients taken in years.	29 to 27
2	Sex	0 for female, 1 for male.	0, 1
3	Chest pain type	There are four types—1 for angina, 2 for atypical angina, 3 for nonangina pain, and 4 for asymptomatic angina.	1, 2, 3, 4
4	Resting blood pressure	Blood pressure of the patient when at rest in mm Hg.	94 to 200
5	Serum cholesterol	The amount of cholesterol in the blood in mg/dL.	126 to 564
6	Fasting blood sugar	Amount of sugar present at fasting. 0 for false—fasting blood sugar is not above 120 mg/dL; 1 for true—fasting blood sugar is above 120 mg/dL.	0, 1
7	Resting electrocardiograph	Values produced by electrocardiography at rest. 0 is normal; 1 is having ST-T wave abnormality; 2 for showing probable or definite left ventricular hypertrophy.	0, 1, 2
8	Maximum heart rate	Maximum heart rate of patient.	71 to 202
9	Exercise-induced angina	Whether or not the patient gets angina when exercise is performed. They are 0 for no and 1 for yes.	0, 1
10	ST depression	Finding on an electrocardiogram wherein the trace of the ST segment is abnormally low below the baseline. Values contain ST depression induced by exercise relative to rest. The abbreviation ST in medical terms means sinus tachycardia.	1 to 3
11	Slope	The slope of the ST segment for peak exercise by the patient. 1 for upsloping, 2 for flat, and 3 for downsloping.	1, 2, 3
12	Number of vessels	Number of vessels colored by fluoroscopy.	0 to 3
13	Thallium stress test result	How well blood flows to the heart while at rest or during exercise. 3 is normal, 6 is a fixed defect, and 7 is a reversible defect.	3, 6, 7
14	Diagnosis	Predicted attribute that contains values showing no presence or presence of heart disease to varying degrees. 0 for no presence, 1 for least likelihood, 2 for moderate likelihood, 3 for a high likelihood, and 4 for very high likelihood. Values 1 through 4 are compressed to a single value, 1, representing the presence of heart disease.	0 or 1

**Table 6:** Interpretation of AUC values.

AUC value	Connotation
0.9 < **AUC** < 1.0	Excellent
0.8 < **AUC** < 0.9	Good
0.7 < **AUC** < 0.8	Fair
0.6 < **AUC** < 0.7	Poor
0.5 < **AUC** < 0.6	Insignificant

## Data Availability

The data for this study are publicly available at https://archive.ics.uci.edu/ml/datasets/heart+disease.

## References

[1] National Center for Health Statistics (NCHS) (2017). *Health, United States, 2016, with Chartbook on Long-Term Trends in Health*.

[2] Murphy S. L., Xu J., Kochanek K. D., Curtin S. C., Arias E. (2017). Deaths: final data for 2015. *National Vital Statistics Reports*.

[3] Benjamin E. J., Virani S. S., Callaway C. W. (2018). Heart disease and stroke statistics-2018 update: a report from the American heart association. *Circulation*.

[4] Roth G. A., Forouzanfar M. H., Moran A. E. (2015). Demographic and epidemiologic drivers of global cardiovascular mortality. *New England Journal of Medicine*.

[5] WHO (2010). *World Health Statistics 2010*.

[6] Bosu W. K. (2013). Accelerating the control and prevention of non-communicable diseases in Ghana: the key issues. *Postgraduate Medical Journal of Ghana*.

[7] Lopez A. D., Mathers C. D., Ezzati M., Jamison D. T., Murray C. J. (2006). Global and regional burden of disease and risk factors, 2001: systematic analysis of population health data. *The Lancet*.

[8] Shah M., Shukla P., Nikam S. (2017). Cardio-vascular disease 9 prediction using genetic algorithm and neuro fuzzy system. *International Journal of Latest Trends in Engineering and Technology*.

[9] Zhang Z., Zhang S., Geng S., Jiang Y., Li H., Zhang D. (2015). Application of decision trees to the determination of the year-end level of a carryover storage reservoir based on the iterative dichotomizer 3. *International Journal of Electrical Power & Energy Systems*.

[10] Shin H. C., Roth H. R., Gao M. (2016). Deep convolutional neural networks for computer-aided detection: CNN architectures, dataset characteristics and transfer learning. *IEEE Transactions on Medical Imaging*.

[11] Onan A. (2020). Sentiment analysis on product reviews based on weighted word embeddings and deep neural networks. *Concurrency and Computation: Practice and Experience*.

[12] Miranda E., Irwansyah E., Amelga A. Y., Maribondang M. M., Salim M. (2016). Detection of cardiovascular disease risk’s level for adults using naive bayes classifier. *Healthcare Informatics Research*.

[13] Karayılan T., Kılıç Ö. Prediction of heart disease using neural network.

[14] Nandy S., Adhikari M., Balasubramanian V., Menon V. G., Li X., Zakarya M. (2021). An intelligent heart disease prediction system based on swarm-artificial neural network. *Neural Computing and Applications*.

[15] Dangare C. S., Apte S. S. (2012). Improved study of heart disease prediction system using data mining classification techniques. *International Journal of Computer Applications*.

[16] Awan S. M., Riaz M. U., Khan A. G. (2018). Prediction of heart disease using artificial neural network. *VFAST Transactions on Software Engineering*.

[17] Sayad A. T., Halkarnikar P. P. (2014). Diagnosis of heart disease using neural network approach. *International Journal of Advances in Science Engineering and Technology*.

[18] Kim J. K., Kang S. (2017). Neural network-based coronary heart disease risk prediction using feature correlation analysis. *Journal of Healthcare Engineering*.

[19] Kweon S., Kim Y., Jang M. J. (2014). Data resource profile: the Korea national health and nutrition examination survey (KNHANES). *International Journal of Epidemiology*.

[20] Dutta A., Batabyal T., Basu M., Acton S. T. (2020). An efficient convolutional neural network for coronary heart disease prediction. *Expert Systems with Applications*.

[21] Singh Y. K., Sinha N., Singh S. K. Heart disease prediction system using random forest.

[22] Javeed A., Zhou S., Yongjian L., Qasim I., Noor A., Nour R. (2019). An intelligent learning system based on random search algorithm and optimized random forest model for improved heart disease detection. *IEEE Access*.

[23] Jabbar M. A., Deekshatulu B. L., Chandra P. (2016). Intelligent heart disease prediction system using random forest and evolutionary approach. *Journal of Network and Innovative Computing*.

[24] Maji S., Arora S. (2019). Decision tree algorithms for prediction of heart disease. *Information and Communication Technology for Competitive Strategies*.

[25] Saxena K., Sharma R. (2016). Efficient heart disease prediction system. *Procedia Computer Science*.

[26] Soleimani P., Neshati A. (2015). Applying the regression technique for prediction of the acute heart attack. *World Academy of Science, Engineering and Technology, International Journal of Medical, Health, Biomedical, Bioengineering and Pharmaceutical Engineering*.

[27] Harimoorthy K., Thangavelu M. (2021). Multi-disease prediction model using improved SVM-radial bias technique in healthcare monitoring system. *Journal of Ambient Intelligence and Humanized Computing*.

[28] Sperandei S. (2014). Understanding logistic regression analysis. *Biochemia Medica: Biochemia Medica*.

[29] Onan A., Korukoğlu S., Bulut H. (2016). A multiobjective weighted voting ensemble classifier based on differential evolution algorithm for text sentiment classification. *Expert Systems with Applications*.

[30] Owusu E., Abdulai J. D., Zhan Y. (2019). Face detection based on multilayer feed‐forward neural network and haar features. *Software: Practice and Experience*.

[31] Mirza I., Mahapatra A., Rego D., Mascarenhas K. Human heart disease prediction using data mining techniques.

[32] Amen K., Zohdy M., Mahmoud M. Machine learning for multiple stage heart disease prediction.

[33] Sajja G. S., Mustafa M., Phasinam K., Kaliyaperumal K., Ventayen R. J. M., Kassanuk T. Towards application of machine learning in classification and prediction of heart disease.

[34] Waris S. F., Koteeswaran S. (2021). Heart disease early prediction using a novel machine learning method called improved K-means neighbor classifier in python. *Materials Today: Proceedings*.

[35] Gupta A., Kumar L., Jain R., Nagrath P. Heart disease prediction using classification (naive bayes).

[36] Saini M., Baliyan N., Bassi V. Prediction of heart disease severity with hybrid data mining.

[37] Abdeldjouad F. Z., Brahami M., Matta N. A hybrid approach for heart disease diagnosis and prediction using machine learning techniques.

[38] Motarwar P., Duraphe A., Suganya G., Premalatha M. Cognitive approach for heart disease prediction using machine learning.

[39] Alotaibi F. S. (2019). Implementation of machine learning model to predict heart failure disease. *International Journal of Advanced Computer Science and Applications*.

[40] Gupta N., Ahuja N., Malhotra S., Bala A., Kaur G. (2017). Intelligent heart disease prediction in cloud environment through ensembling. *Expert Systems*.

